# Age and Sex Variation in the Duration of Action and Corneal Touch Threshold (CTT) following Instillation of 0.5% Topical Ophthalmic Proparacaine and Tetracaine Hydrochlorides

**DOI:** 10.1155/2021/8661098

**Published:** 2021-07-14

**Authors:** Samuel Kyei, Nana Yaw Abaka Dadzie, Ebenezer Zaabaar, Kwasi Antwi Asamoah Dwomoh, Kofi Asiedu

**Affiliations:** ^1^Department of Optometry and Vision Science, School of Allied Health Sciences, College of Health and Allied Sciences, University of Cape Coast, Cape Coast, Ghana; ^2^The Eye Clinic, LEKMA Hospital, Teshie, Accra, Ghana; ^3^School of Optometry and Vision Science, University of New South Wales, Sydney, Australia

## Abstract

**Purpose:**

We investigated the effect of age and sex on corneal touch threshold (CTT) and duration of action following administration of 0.5% topical ophthalmic proparacaine and tetracaine hydrochlorides.

**Methods:**

A prospective, randomized, subject-masked, crossover study design was used. Two hundred and forty human volunteers were enrolled in the study. Corneal touch threshold (CTT) was determined using a Cochet-Bonnet esthesiometer. CTT was measured every 15 seconds for the first 1-minute and at 5-minute intervals subsequently for a period of 40 minutes after the application of each anesthetic. CTT and duration of action of the ophthalmic solutions were tested for statistical significance using repeated measures ANOVA.

**Results:**

The total duration of effect was 20 minutes for females and 25 minutes for males for both anesthetics. The total duration of the effect of both solutions decreased with increasing age; however, elderly participants had the longest duration (5 minutes) of the maximal effect (minimum CTT) of the two ophthalmic preparations. There was a significant influence of sex, F (2.39, 569.65) = 2.86, *p*=0.04; F (3.48, 828.19) = 4.41, *p*=0.003, and age, F (4.78, 566.18) = 8.97, *p* < 0.001; F (7.19, 852.56) = 20.55, *p* < 0.001 on CTT following application of proparacaine hydrochloride and tetracaine hydrochloride, respectively.

**Conclusion:**

CTT and duration of anesthetic effect after instillation of 1 drop of 0.5% proparacaine hydrochloride and 0.5% tetracaine hydrochloride vary based on sex and age.

## 1. Introduction

Assessment of corneal touch threshold (CTT) using the esthesiometer has been studied in corneal pathologies, preoperative and postoperative corneal surgeries, to verify predictive sensibility deficits of unrecognized pathologies [1, 2]. The corneal sensibility and the duration of the anesthesia are therefore fundamental in anterior segment surgeries, especially in complex interventions involving the cornea and the lens [1, 2].

Anesthetics are medications that block sensation, and they work by blocking neural impulses [3]. Local anesthetics are primarily weak bases that are made up of three important components: an aromatic ring, an intermediate-length ester or amide linkage, and a tertiary amine [4]. These components contribute to their lipid solubility, the extent of protein binding, and dissociation constant (pKa) [5–10], which are also correlated with their potency, duration of action, and onset, respectively [11, 12].

Age and sex are two of the most important demographic characteristics of patients regarding clinical practice [13]. Aging is associated with physiologic changes which could alter the pharmacokinetics of drugs. Age-related changes such as an increase in body fat, decrease in lean body mass, the affinity of serum protein to protein-bound drugs, and decrease in total body water affect drugs absorption, distribution, metabolism, and elimination [14–20]. Oertel et al. [21] reported a trend of higher serum concentration of a local anesthetic (articaine) in older healthy subjects versus young participants. The authors attributed this finding to lean body mass, increase in body fat, and decrease in total body water in the elderly.

The physiological differences between males and females, which impact pharmacokinetic parameters, have also been extensively described in some reviews [22, 23]. In humans, it is estimated that there is a 40% difference in pharmacokinetics between males and females [13]. The female sex hormone, progesterone, has been shown to increase the potency of inhaled anesthetics [24]. Besides, altered modulation of the *γ*-hydroxybutyric acid receptor complex, one of the purported sites of action of anesthetic drugs, has been demonstrated during the menstrual cycle in rats [25]. Previous studies [26] suggest that women recover faster from general anesthetic drugs and are eligible for discharge from the recovery room sooner than men. The authors attributed this finding to a possible effect of female sex hormones on the modulation of anesthetic actions. Information on the effect of sex disparities on local anesthetics is very scanty and does not permit any conclusion.

From the foregoing perspective, different sex and age categories could have different responses to the commonly used topical ophthalmic proparacaine and tetracaine since these preparations bind to proteins and exist in both ionized (water-soluble) and unionized (lipid-soluble) forms [27]. Studies have demonstrated the action and duration of tetracaine and proparacaine in dogs, cats, rabbits, horses, and humans [28–32]. However, to the best of our knowledge, no studies have investigated the influence of age and sex disparities on corneal touch threshold (CTT) and duration of effect following instillation of these anesthetics. Most practitioners do not even consider age and sex in the administration of anesthetics and may cause discomfort to the patient during and after procedures. Therefore, the objective of the study was to determine the effect of age and sex disparities on CTT and duration of action following the instillation of topical ophthalmic tetracaine and proparacaine in humans.

## 2. Materials and Methods

A prospective, randomized, subject-masked, crossover study design was used. Two commercially available anesthetic ophthalmic solutions containing 0.5% proparacaine hydrochloride and 0.5% tetracaine hydrochloride were used. Subjects were randomly assigned to receive either 0.5% proparacaine hydrochloride or 0.5% tetracaine hydrochloride on the first visit, and after a one-week washout phase, the process was repeated until all participants had received both anesthetics. Treatment consisted of topical instillation of 1 drop of the ophthalmic anesthetic agent in the lower conjunctival cul-de-sac. The same investigator performed all treatments and measurements.

Data collection was done at the University of Cape Coast Eye Clinic. Subjects from the University community were recruited into the study. A total of 240 healthy individuals volunteered to participate in the study after discussing the specifics and possible consequences of the study to them. Participants were categorized into young (18–35), middle-aged (36–55), and elderly (above 55). Participants were excluded if they had diabetes, hypertension, a history of corneal disease, or evidence of other known pathologies that could impair the sensitivity of the cornea.

The study was approved by the institutional review board (IRB) of the University of Cape Coast, Ghana (UCCIRB/CHAS/2018/56), and followed the tenets of the Declaration of Helsinki. Experimental procedures were conducted following good clinical practice guidelines, and all data and records generated throughout the course of the study were handled with strict confidentiality per the University of Cape Coast institutional policy. Informed consent was obtained from each participant before the commencement of data collection.

The participants were given 1 drop of the study solutions in the lower conjunctival cul-de-sac. A Cochet-Bonnet esthesiometer with a 0.12 mm cross-sectional diameter nylon monofilament was used to measure the CTT, the minimal amount of corneal stimulation that results in a blink reflex. All CTT recordings were obtained from the central cornea. The length of the nylon filament of the esthesiometer could be varied and directly corresponded to the touch pressure applied to the cornea. The first touch was made with the longest thread length of 6 cm. If there was no response, the filament length was decreased in 0.5 cm steps until the subject had a consistent corneal blink reflex in response to the corneal stimulus. Blinks stimulated by unintentional contact with eyelashes were excluded. When a corneal blink reflex was elicited, the length of the nylon filament was recorded as the CTT. The CTT of the eye was recorded as 0 cm if there was no blink response with the shortest filament length of 0.5 cm.

The baseline CTT of each participant was measured within 30 minutes before the administration of the anesthetic solution. Time 0 (T0) was recorded as the time at which baseline measurements were taken. The CTT was measured every 15 sec for the first minute (T1) after application of 1 drop of the anesthetic and at 5-minute intervals subsequently for a period of 40 minutes after application, to determine changes in corneal sensitivity across time.

Repeated measures ANOVA was performed using IBM SPSS Statistics for Windows, Version 22.0, Armonk, NY : IBM Corp, and a *p* value of <0.05 was considered statistically significant. Greenhouse-Geisser corrected degrees of freedom were computed to minimize the effects of violating assumptions about data sphericity by repeated measures ANOVA. Post hoc Tukey HSD test was used when applicable.

## 3. Results

**Figure 1:**
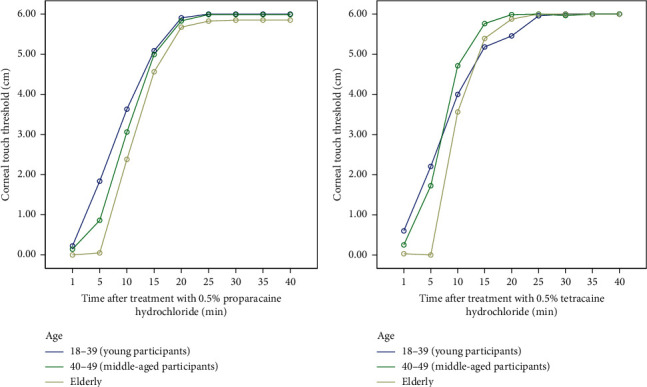
Time course curve showing the effect of age on the onset and duration of actions of (a) proparacaine and (b) tetracaine. Values plotted are mean corneal touch threshold ± standard error of mean (*n* = 80). Repeated measures ANOVA followed by post hoc Tukey HSD.

**Figure 2:**
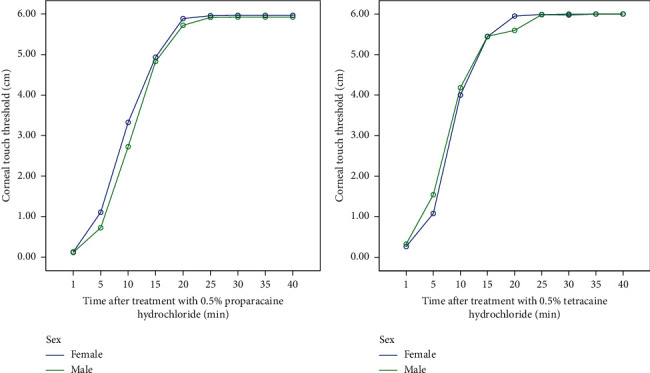
Time course curve showing the effect of sex on the onset and duration of actions of (a) proparacaine and (b) tetracaine. Values plotted are mean corneal touch threshold ± standard error of mean (*n* = 120). Repeated measures ANOVA followed by post hoc Tukey HSD.

All 240 participants enrolled in the study completed both sessions of the study. One hundred and twenty, 120 (50%), were males and 120 (50%) were females. There were 80 participants in each of the young, middle-aged, and elderly groups. The mean baseline sensitivity was 5.84 ± 0.58 (95% CI, 5.76–5.91). The minimum mean CTT values and total duration of action of the two solutions are summarized in Table 1 and Figure 1 for age groups and in Table 2 and Figure 2 for gender categories. As shown in Figure 1, the maximal effect of both anesthetics remained relatively constant for 5 minutes in the elderly.

### 3.1. Effect of Age and Sex on the Onset and Duration of Proparacaine Hydrochloride

There was a significant effect of sex on CTT after application of proparacaine, *F* (2.39, 569.65) = 2.86, *p*=0.04; similarly, the effect of age on CTT after application of proparacaine was significant, *F* (4.78, 566.18) = 8.97, *p* < 0.001. Post hoc comparisons using the Tukey HSD test indicated that CTT in elderly participants after application of proparacaine was significantly lower when compared to those of young participants (MD = 0.52 SE = 0.08), *p* < 0.001, and middle-aged participants (MD = 0.31, SE = 0.08), *p*=0.001.

### 3.2. Effect of Age and Sex on the Onset and Duration of Tetracaine Hydrochloride

There was a significant effect of sex on CTT after application of tetracaine, *F* (3.48, 828.19) = 4.41, *p*=0.003; similarly, the effect of age on CTT after application of tetracaine was significant, *F* (7.19, 852.56) = 20.55, *p* < 0.001.

Post hoc comparisons using the Tukey HSD test indicated that CTT in elderly participants after application of tetracaine did not differ significantly when compared to those of young participants (MD = 0.15, SE = 0.08), *p*=0.12, but was significantly lower when compared to middle-aged participants (MD = 0.39, SE = 0.08), *p* < 0.001.

## 4. Discussion

The time of onset of both anesthetics was within 1 min for all age and sex categories. The results of the present study indicate that application of one drop of 0.5% proparacaine hydrochloride results in a comparatively low minimum CTT in males than in females. Contrarily, the results show that a drop of 0.5% tetracaine hydrochloride produces a relatively low minimum CTT in females compared to males.

Minimum CTT values decreased with increasing age for both ophthalmic solutions. Both anesthetics had the longest total duration of effect in males than females. The total duration of the effect of both proparacaine and tetracaine decreased with increasing age. The duration of maximal effect (minimum CTT) was within 1 min for young and middle-aged participants but lasted for 5 min for elderly participants.

Mean baseline central CTT did not differ substantially from that of a previous study [33], in which the mean baseline threshold of corneal sensitivity of healthy human subjects was 5.85 cm. Objective studies on the onset and duration of corneal anesthesia following topical administration of 0.5% tetracaine in humans are limited.

In a study conducted on 23 clinically normal human subjects at the Albany Medical Center in New York [27], the effects of 0.5% proparacaine and 0.5% tetracaine lasted for 10.7 min and 9.4 min, respectively. The authors evaluated corneal sensitivity with a cotton wisp and a subjective visual-analog pain rating scale; this together with the smaller sample size could account for the shorter total duration of the effect reported in the study. Weiss and Goren [33] reported a complete recovery time of 34.86 min in 7 clinically normal control eyes after treatment with 0.5% proparacaine. The authors reported that the duration of maximal effect (minimum CTT) averaged 11.71 min. The difference in the duration of maximal effect, as well as the total duration of action between the two studies, could be due to variability in sample size and the intervals at which CTT was measured within the first 5 min.

In the present study, the comparatively long duration of the maximal effect (minimum CTT) of the anesthetics in the elderly could be related to increased body fat, reduced lean body mass, and decreased total body water associated with aging. Moreover, the relatively short total recovery time of the anesthetics in the elderly could be attributed to the less affinity of proteins to protein-bound drugs in old age. Additionally, the underlying physiological changes induced by sex hormones could account for the sex variability in the total duration of the ophthalmic solutions.

We recommend that further studies be conducted to investigate and validate the roles of sex hormones and protein binding in sex and age differences associated with the onset, potency, and duration of topical ophthalmic proparacaine and tetracaine. The current study suggests that topical 0.5% proparacaine hydrochloride and 0.5% tetracaine hydrochloride may have to be readministered in female and elderly patients during diagnostic or therapeutic procedures in which a longer duration of reduced corneal sensitivity is required. Furthermore, the longer duration of maximal effect among elderly participants may result in corneal injury during procedures such as contact tonometry due to decreased corneal protection through inhibition of the corneal reflex as well as decreased tear production.

The limitation of this study is that the investigator was not masked to the ophthalmic solutions applied. In conclusion, we investigated the influence of age and gender disparities on duration and CTT following the instillation of topical ophthalmic proparacaine hydrochloride and tetracaine hydrochloride in clinically healthy humans. The effect of both anesthetics lasted longer in males than females and longer in young and middle-aged participants than elderly participants. Mean CTT values significantly differed with gender and age, and minimum CTT decreased with increasing age.

## Figures and Tables

**Table 1:** Minimum CTT values and total duration of effect with respect to age.

Anesthetic	Age group	Minimum CTT (cm)	Total duration (min)
Proparacaine HCL	Young	0.23 ± 0.71	25
	Middle-aged	0.14 ± 0.65	25
	Elderly	0.00 ± 0.00	20

Tetracaine HCL	Young	0.60 ± 1.23	30
	Middle-aged	0.25 ± 0.91	20
	Elderly	0.00 ± 0.00	20

**Table 2:** Minimum CTT values and total duration of effect with respect to sex.

Anesthetic	Sex	Minimum CTT (cm)	Total duration (min)
Proparacaine HCL	Female	0.13 ± 0.60	20
	Male	0.12 ± 0.52	25

Tetracaine HCL	Female	0.26 ± 0.93	20
	Male	0.33 ± 0.96	25

## Data Availability

The data used to support the findings of this study are included within the article.
